# Hepatitis E Virus infection in HIV-infected patients with elevated serum transaminases levels

**DOI:** 10.1186/1743-422X-8-171

**Published:** 2011-04-15

**Authors:** Pierre Sellier, Marie-Christine Mazeron, Sophie Tesse, Esma Badsi, John Evans, Jean-Dominique Magnier, Marie-Jose Sanson-Le-Pors, Jean-François Bergmann, Elisabeth Nicand

**Affiliations:** 1 Unite des Maladies Infectieuses et Tropicales, Service de Medecine Interne A, Hopital Lariboisiere, 2, rue Ambroise Pare, 75010, Paris, France; 2 Laboratoire de Bacteriologie-Virologie, Hopital Lariboisiere, 2, rue Ambroise Pare, 75010, Paris, France; 3 CNR Virus de l'hepatite E, Service de Biologie Medicale, Hopital d'Instruction des Armees du Val-de-Grace, 74, boulevard de Port-Royal, 75230, Paris, France; 4 immeuble SCOR, 1, avenue du General de Gaulle, 92074, Paris la Defense, France

## Abstract

Increases in aminotransferases levels are frequently encountered in HIV-positive patients and often remain unexplained. The role in this setting and natural history of hepatitis E in HIV-infected patients are unknown. The aim of the study was to assess HEV infection in HIV-infected patients attending a Parisian hospital, with a current or previous cryptogenic hepatitis.191 plasma samples collected from 108 HIV-infected patients with elevated aminotransferases levels were retrospectively tested for the presence of hepatitis E virus (HEV) infection markers: anti-HEV IgM antibodies, anti-HEV IgG antibodies, anti-HEV IgG avidity index and plasma HEV RNA.One acute infection, documented by positive tests for anti-HEV IgM antibody, low anti-HEV IgG avidity index and plasma HEV RNA (genotype 3e), and three past infections were diagnosed, without any observed case of persistent infection. The acute hepatitis was benign and resolved spontaneously within two weeks. This infection was probably contracted locally. Acute HEV hepatitis can occur in HIV-infected patients but rarely explains cryptogenic hepatitis, at least in an urban HIV population, regardless geographic origin and CD4 counts.

## Findings

Hepatitis E virus (HEV) hepatitis is endemic in developing and emerging in industrialized countries [1], where seroprevalence ranges from 1 to 20% [2]. HEV was thought to cause acute hepatitis, but chronic hepatitis in organ transplant recipients [3], and reactivation after stem cell transplantation [4] have been reported. Few acute infections [5,6] and a persistent carriage [7] in HIV-positive patients have been published. As elevated transaminase levels are frequent, often unexplained in HIV-positive patients, we studied the role of HEV in this setting.

From 1250 HIV-positive patients followed-up in the Infectious Diseases Department, 108 with at least one episode of elevated aminotransferase levels above twice the upper limit of normal (ULN, 50 I.U./mL) between January 2005 and December 2008 were included after written consent was obtained. As hepatitis E can worsen chronic liver disease [8], and be misdiagnosed with drug-induced liver injury [9], HBsAg or HCV RNA-positive patients, those with alcoholic or drug-induced liver injury were not excluded.

Plasma was screened retrospectively for anti-HEV IgG and IgM (EIAgen HEV IgG^®^, EIAgen HEV IgM^®^, Adaltis, Bologna, Italy), based on synthetic immunodominant determinants encoded by ORF2 (aa 619-660) and ORF3 (aa 101-123) derived from Burma virus and Mexican strain. From 200 μl of plasma, HEV RNA was amplified, using real-time RT-PCR able to amplify any HEV genotype with a limit detection test of 500 copies/ml [10]. An external inhibition control was tested for each sample to rule out possible inhibitors with calcium ions containing in EDTA tubes used for collection of plasma. For IgG positive samples, IgG avidity index was determined to differentiate recent (avidity index< 40%) from past infection (avidity index> 40%), this test being previously validated [11].

From 108 included patients (M/F: 2.3, ages: 42.1 ± 8.6 years for males, 38.3 ± 9.5 years for females), two hundred and twelve episodes of elevated transaminases levels were recorded (1 to 8/patient), from which 191 plasma (1 to 8/patient) could be tested. CD4 count was 347 ± 225/mm^3 ^and HIV RNA load was 5.3 ± 6 log_10_/mL at the onset of transaminasitis; 86/108 patients were given antiretroviral therapy (ART), 18/108 (16.7%) were HBV, 25/108 (23.1%) were HCV, 3/108 (2.8%) were HBV-HCV-coinfected respectively.

Acute HEV infection was diagnosed in one patient (Table, Patient 1). He was born in France, homosexual, tested HIV-1 positive in 2006, with 340 CD4/mm^3 ^and 7,000 copies/mL. Prophylaxis with trimethoprim/sulfamethoxazole was begun in April 2008 (280 CD4/mm^3^, 12%). In June, ART (tenofovir/emtricitabine + atazanavir/ritonavir) was started; biological liver tests were normal. Eight weeks later, alanine (ALT) and aspartate (AST) aminotransferases reached respectively 20 ULN and 12 ULN, without any physical complaints. ART was withdrawn, biological tests normalized within two weeks. HEV RNA (genotype 3e, Genbank GU084155), anti-HEV IgM and IgG (avidity index 10%) were present, confirming a recent infection. Hepatitis A, B, C acute infections were excluded. HEV infection was self limiting, with no persistent carriage. The original ART schedule was resumed, without any episode of transaminasitis. Neither HEV RNA nor anti-HEV antibodies were detected three weeks prior to the onset of hepatitis, showing recent exposure to HEV. The patient denied travel to endemic regions but reported regular consumption of undercooked pork. His partner was tested negative for serological and molecular HEV markers (Table 1).

**Table 1:** Demographic and biological characteristics of patients seropositive for HIV-1 with acute or past HEV infection

**Patient N**_**°**_	Sex	Age (years)	ALT (xULN)	AST (xULN)	**CD4 + T lymphocytes absolute count/mm**^**3**^	**Plasmatic HIV RNA (log**_**10 **_**copies/mL)**	**Sample N**_**°**_	Time from onset of transaminasitis (months)	Anti HEV IgM OD/CO	Anti HEV IgG OD/CO	IgG avidity index (%)	RNA HEV	Conclusion
					**At onset of transaminasitis**								
1	M	34	1	1			1	- 1	0.5	0.6		NEG	No HEV infection
			20	12	286	3.4	2	0	10.8	4.2	7.5		Acute HEV
			1	1			3	+1	10.8	4.2	11	POS	infection
			1	1			4	+12	2.2	3.9	34		
												NEG	
												NEG	
2	F	32	8	3	215	4.1	1	0	0.1	4.3	86	NEG	Past infection
3	M	57	2.5	2.5	223	5.1	1	0	0.2	7.2	73	NEG	Past infection
4	M	52	2	2.5			1	- 4	0.7	4.8	90	NEG	Past infection
			4	3	246	3.0	2	0	0.9	5.4	88		
			1	1			3	+ 12	0.7	7.1	93	NEG	
			1	1			4	+ 18	0.6	7.0	100	NEG	
												NEG	

OD/CO: Optical density/Cut Off; the results were considered as positive if OD/CO exceeded 1.

ULN: Upper Limit of Normal values, 50 I.U./mL. ALT: alanine aminotransferases. AST: aspartate aminotransferases.

Past HEV infection was diagnosed in three patients, based on detection of IgG without IgM, and negative RNA. The first case (Table, patient 2), born in Cameroon, had an acute episode of fever of unknown origin with transaminasitis. She reported frequently eating undercooked bush red meat (braised monkey, coypu, wart hog, porcupine meat) in Cameroon. The second case (Table, patient 3) was detected in a man born in Yugoslavia. Increase of aminotransferases was related to myocardial infarction. He mentioned eating undercooked pork meats coming from cattle farms in Yugoslavia during childhood. The third case (Table, patient 4) was reported in a patient born and living during his childhood in Algeria. Chronic transaminasitis was attributed to HCV-coinfection.

Markers of HEV infection were detected in four (3.7%) of the HIV-infected patients with elevated transaminase levels. One acute indigenous infection was diagnosed, with a favourable outcome, as in few previously reported cases [5,6], whose CD4 were above 200/mm^3^, contrasting with a persistent carriage in a HIV-infected patient with CD4 below 200/mm^3 ^[7], and chronic HEV hepatitis in organ-transplant recipients [3] and patients with leukaemia [4]. Avidity maturation process is T-cell dependent and CD4 are the lines of specific immune response to HEV [12]. In our patient, IgG avidity index remained low after one year, suggesting a low maturation of immune response. Correspondingly, a defect in specific antibody avidity in response to measles vaccination and to measles was reported in HIV-infected children [13].

The source of indigenous hepatitis E infection in industrialized countries is uncertain. In our case of acute infection, the hypothesis of sexual transmission from his partner can be discarded, as he was tested negative for HEV.

In three patients with past infection, the exposure may have been in their native countries, the date of contamination could not be determined.

HEV was possibly transmitted in these four patients via consumption of undercooked pork, but there is no control group and no HEV RNA detection from the potential source was possible in our study.

Epidemiologic studies of HEV hepatitis in HIV-infected patients are scarce. In Buenos Aires, the seroprevalence (6.6%) was higher than in control groups (1.8%), most of the patients being intravenous drug users or homosexuals [14]. Recently, Madejon *et al*. have investigated 50 HIV-infected patients with CD4 < 200 cells/mm^3 ^and 43 with cryptogenic hepatitis, and did not found HEV viremia [15]. Renou *et al*. identified three Caucasian patients with acute HEV infection from a survey conducted in Southern France which screened 133 patients [6]. As with the two previous reports [6,15], this present study, testing patients with elevated transaminases, was not designed to estimate the prevalence of anti-HEV in the HIV-infected patients.

Testing 191 plasma samples in 108 patients with at least one episode of elevated aminotransferase levels during four consecutive years, we described a single case of indigenous acute HEV hepatitis. Acute hepatitis can occur in HIV-infected patients but rarely explains cryptogenic hepatitis, at least in an urban population, regardless of geographic origin and CD4 counts. Nevertheless, with the increased number of indigenous Hepatitis E infection in France (a total of 180 cases in 2009 in France at least (unpublished data from CNR Hepatitis E virus)) the investigation of HEV markers in HIV-infected patients with unexplained elevated transaminases should be recommended.

## Competing interests

The authors declare that they have no competing interests.

## Authors' contributions

PS, MCM, EN contributed to study concept and design. JDM was responsible for the statistical analysis. ST, EB, JE revised the manuscript for intellectual content. All authors read and approved the final version of the manuscript.
